# Investigation of a Carbapenemase-producing *Acinetobacter baumannii* outbreak using whole genome sequencing versus a standard epidemiologic investigation

**DOI:** 10.1186/s13756-018-0437-7

**Published:** 2018-11-21

**Authors:** Chloe Bogaty, Laura Mataseje, Andrew Gray, Brigitte Lefebvre, Simon Lévesque, Michael Mulvey, Yves Longtin

**Affiliations:** 1 University of Manitoba, Winnipeg, MB Canada; 2 National Microbiology Laboratory, Winnipeg, MB Canada; 3 Northern Health, Prince George, BC Canada; 4 Laboratoire de santé publique du Québec, Sainte-Anne-de-Bellevue, QC Canada; 5 McGill University, Montreal, QC Canada

**Keywords:** Outbreak investigations, Whole-genome sequencing, Acinetobacter baumannii, Carbapenemase-producing organisms, Single nucleotide variant analysis

## Abstract

**Background:**

The standard epidemiologic investigation of outbreaks typically relies on spatiotemporal data and pulsed-field gel electrophoresis (PFGE), but whole genome sequencing (WGS) is becoming increasingly used. This investigation aimed to characterize a carbapenemase-producing *Acinetobacter baumannii* (CPAb) nosocomial outbreak using WGS compared to a standard outbreak investigation.

**Methods:**

The CPAb outbreak occurred in a single center between 2012 and 2014. The standard investigation used spatiotemporal data and PFGE to generate a chain of transmission. A separate WGS investigation generated a chain of transmission based solely on WGS and date of sampling and was blinded to all other spatiotemporal data and PFGE. Core single nucleotide variant (SNV) phylogenetic analysis was performed on WGS data generated using the Illumina MiSeq platform. The chains of transmission were compared quantitatively and qualitatively to assess the concordance between both methods.

**Results:**

28 colonized and infected cases were included. Of the 27 transmission events identified using the standard investigation, 12 (44%) were identical to the transmission events using WGS. WGS identified several transmission events that had not been detected by traditional method, and numerous transmission events that had occurred on different hospital wards than suspected by standard methods. The average number (standard deviation [SD]) of SNVs per transmission events was 1.63 (SD, 1.31) by traditional method and 0.63 (SD, 0.79) by WGS (*p* = 0.001) All isolates harbored the rare carbapenemase *bla*_OXA-237_.

**Conclusions:**

The traditional and WGS investigations had moderate concordance. When used alongside epidemiologic data and clinical information, WGS could help improve the mapping of transmission events.

## Background

Carbapenemase-producing *Acinetobacter baumannii* (CPAb) is of global concern [1]. Canada has had infrequent cases [2], but has not been spared from the occasional outbreak [3]. The most recent reported outbreak occurred from 2012 to 2014 in a primary and tertiary institution in Montreal (Canada) [4].

Control of CPAb outbreaks requires meticulous investigation. A standard epidemiologic investigation is a multi-step process including the creation of a chain of transmission [4]. Phenotypic characteristics such as biotypes, serotypes, and antimicrobial susceptibilities are used to infer pathogen inter-relatedness and is combined with spatiotemporal information to determine transmission events [5]. This information is sometimes combined with molecular typing methods (commonly pulsed-field gel electrophoresis (PFGE)). PFGE is usually considered the gold standard for its ability to detect differences across the entire genome [5, 6].

Whole genome sequencing (WGS) is increasingly being recognized as a powerful tool for epidemiologic studies. Its ability to distinguish strains that differ by single nucleotides provides high levels of resolution [7, 8]. WGS has been employed in outbreak investigations, both retrospectively to identify environmental sources [9] and additional cases [10], but also in real-time to confirm the existence of an outbreak [11] and track the emergence and spread of mutational resistance mechanisms [12]. However, only a few studies have compared standard epidemiologic outbreak investigations with investigations performed by WGS. Through the subsequent use of WGS, Kanamori et al observed that two epidemiologically unlinked outbreaks were in fact genetically connected [13]. In another study, WGS allowed for the identification of unexpected transmission routes not initially suggested by the epidemiologic data [14].

The aim of this study was to contrast the validity and accuracy of a standard epidemiologic outbreak investigation with one achieved using WGS. We retrospectively characterized the CPAb nosocomial outbreak that occurred in an academic institution in Montreal (Canada) using only WGS analysis and compared the predicted chain of transmission with an existing standard epidemiologic investigation.

## Methods

### Setting and definitions

A CPAb outbreak occurred in a 637-bed primary and tertiary care hospital between March 2012 and January 2014 [4]. A confirmed case was defined as any patient found to be colonized or infected during this period by the outbreak strain, as confirmed by pulsed-field gel electrophoresis interpretative criteria [15].

### Standard epidemiologic investigation

Screening was performed using Acinetobacter-selective chromogenic agar (CHROMagar Acinetobacter, CHROMagar Microbiology, Paris, France). While initially carried out in patients who had shared hospital rooms with known cases, screening eventually evolved to include all patients sharing a ward with a case. Organism identification was conducted using matrix-assisted laser desorption/ionization time-of-flight mass spectrometry (MALDI-TOF MS, bioMérieux, Marcy l’Étoile, France) and antimicrobial susceptibility testing with disc diffusion and E-test following the Clinical and Laboratory Standards Institute breakpoints (2014 M100S). Strain clonality was confirmed by PFGE. Bacterial genomes were digested with *Apa*I restriction enzyme and samples were run on a CHEF-DR III system (Bio-Rad, Mississauga, ON, Canada). Banding patterns were analyzed using Tenover’s criteria [15]. Detection of resistance genes was performed at the National Microbiology Laboratory in Winnipeg (Canada) [16]. Determining the chain of transmission followed a previously described approach, using a combination of spatiotemporal patient information, date of strain isolation, antibiogram results, and PFGE interpretations [4].

### DNA extraction and whole genome sequencing

Total cellular deoxyribonucleic acid (DNA) was prepared using Epicentre MasterPure™ Complete kits (Mandel Scientific, Guelph, ON). Libraries were created with TruSeq Nano DNA HT sample preparation kits (Illumina, San Diego, CA). Paired-end, 301 bp indexed reads were generated on an Illumina MiSeq™ platform (Illumina, San Diego, CA). De novo assembly of Illumina reads were done using Spades v3.5. Assembled sequence data was analysed using the batch uploader mode at the Centre for Genomic Epidemiology website (https://cge.cbs.dtu.dk/services/) and data was produced from the ResFinder, PlasmidFinder, VirulenceFinder, and MLST tools.

### Single nucleotide variant (SNV) analysis and creation of WGS-based chain of transmission

SNV analysis was conducted using a previously published pipeline [17]. The following parameters were added: map/base quality 30, alternate fraction 0.75, minimum read coverage of identification of variants 10, SNV density filtering of 2 SNVs within a 20-base pair window. The reference strain used was an assembled genome of one of the internal isolates to the outbreak (patient 29). Maximum likelihood phylogenetic trees were built using PhyML v3.1 using parameters “—quiet –b − 4 –m GTR –s BEST” [18]. Images were rendered in FigTree (v1.4.1) (http://tree.bio.ed.ac.uk/software/figtree).

A WGS-based chain of transmission was generated using only the sequencing data and the date of sampling to determine directionality, with complete blinding of the epidemiologic data. A cut-off of ≤2 SNV was selected to indicate a putative transmission event between patients. Also, when > 1 potential source patient was plausible for a given transmission event, transmission events with a lower number of SNVs were considered more likely (e.g. 0 SNV more likely than 2 SNVs).

### Comparison of the outbreak investigation methods

To compare the chains of transmission generated by standard epidemiologic investigation vs. WGS, four different strategies were used. Firstly, both chains of transmission were compared qualitatively. For each transmission event, epidemiologic data was revised to assess where patients had likely acquired the outbreak strain. Secondly, WGS was used to verify the plausibility of each transmission event determined by the standard investigation. Transmission events that remained unchanged were deemed to be supported by WGS, while those that differed were considered unsupported. Thirdly, quantitative SNV differences were calculated by comparing the mean number of SNVs per transmission event between the two investigation methods using the Student’s T-test. Statistical significance was defined as a *p*-value of < 0.05. Finally, the WGS-generated data was compared to that obtained by PFGE for each isolate.

## Results

### Outbreak description

Twenty-eight cases were identified during the outbreak. The index case occurred in the intensive care unit in March 2012. Twenty-seven additional cases were detected up to 2014. Five cases developed CPAb bacteremia, and all died within 72 h of the first positive blood culture. These represented the only cases of infection, while the remaining 22 cases were only colonized. All isolates were resistant to penicillins, carbapenems, quinolones, and aminoglycosides. Four pulsovars were identified by PFGE. Further details on patient characteristics and the course of the outbreak have been reported previously [4].

### Genomic analysis

By in silico sequence typing, all 28 isolates were found to be of sequence type (ST) 208. Resistant genes identified by WGS were consistent for all isolates. Carbapenem resistance was attributable to the presence of the acquired class D carbapenemase *bla*_OXA-237_ as well as the intrinsic *bla*_OXA-66_. Aminoglycoside resistance was attributable to the presence of *aadA1*, *aph(3′)-Ia*, *armA*, *strA* and *strB*. Quinolone resistance was due to mutations in both GyrA (Ser-83-Leu) and ParC (Ser-80-Leu) [19, 20].

The total percentage of valid and included positions used for SNV analysis represented 96.8% of the total core genome. A total of 20 SNVs were used to generate phylogeny as they were identified across all outbreak strains. These 20 SNV loci were extracted and aligned for comparison (Table 1).

**Table 1:** Single nucleotide variant (SNV) differences between outbreak strains

			SNV Analysis	
Year	Identifier	PFGE Pulsovar	Pseudo-alignment	Difference in SNVs from Index Case
2012	1	A5-c	CGCAAGCTTCTGGTTTAACC	Index case
	2	A	C**A**CAAG**AC**TCTGGTT**C**AACC	4
	3	A5	CGCAAG**AC**TCTGGTTTAACC	2
	4	A4	CGC**C**AGCTTCTG**A**TTTAACC	2
2013	5	A5	CGCAAG**AC**TCTGGTTTAACC	2
	6	A5	CGCAAG**AC**TC**C**GGTTTAACC	3
	7	A5	CGCAAG**AC**TC**C**GGTTTAACC	3
	8	A5	CGCAAG**AC**TC**C**GGT**G**TAACC	4
	9	A5	CGCAAG**AC**TC**C**GGTTTA**G**CC	4
	10	A5	CGCAAG**AC**TC**C**GGTTTAACC	3
	11	A5	CGCAAG**ACC**CT**A**GTTTAACC	4
	12	A5	CGCAAG**AC**TC**C**GGTTTAACC	3
	13	A4	CGCA**TAAC**TC**C**GGTTTAACC	5
	14	A6	CGCAA**AAC**TC**C**GGTTTAACC	4
	15	A4	CGCA**TAAC**TC**C**GGTTTAACC	5
	16	A6	CGCA**TAAC**TC**C**GGTTTAACC	5
	17	A5	CGCAAG**AC**TCTGGTTTAACC	2
	18	A5	**T**G**T**AAG**AC**TCTGGTTTAACC	4
	19	A5-a	CGCAAG**AC**TCTGGTTTAACC	2
	20	A5-a	CGCAAG**AC**TCTGGTTTAACC	2
	21	A5-a	CGCAAG**AC**TCTGGTTTAACC	2
	22	A5-a	CGCAAG**AC**TCTGGTTTAACC	2
	23	A5-a	CGCAAG**AC**TCTGGTTTAACC	2
	24	A5	**T**G**T**AAG**AC**TCTGGTTTAACC	4
	25	A5	CGCAAG**AC**TCTGGTTTAA**T**C	3
	26	A5	**T**G**T**AAG**AC**TCTGGTTTAACC	4
	27	A5	**T**G**T**AAG**AC**TCTGGTTTAACC	4
2014	28	A6	CGCAAG**AC**TCTGG**A**TT**C**ACC	4

Bolded letters represent a SNV difference compared to the index case

Abbreviations: *SNV*, single nucleotide variants; *PFGE*, pulsed-field gel electrophoresis; *Q*, quarter

### Comparison of epidemiologic investigations

**Fig. 1:**
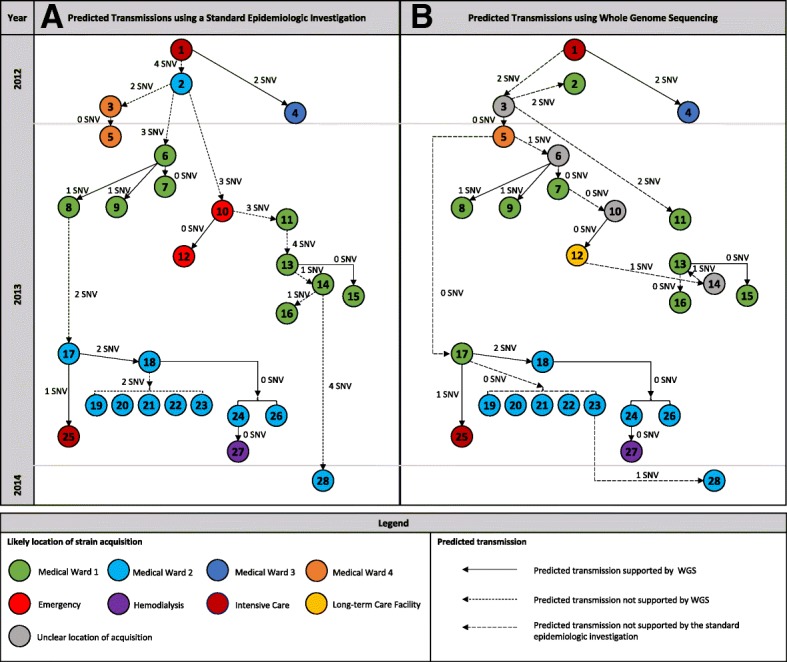
Transmission of a carbapenemase-producing *Acinetobacter baumannii* during a hospital outbreak in Montreal, Canada (axis not to scale). (**a**) Standard epidemiologic investigation, where predicted transmissions were determined using spatiotemporal patient information, date of strain isolation, antibiogram results, and pulsed-field gel electrophoresis interpretation. (**b**) Whole genome sequencing investigation, where predicted transmissions were determined using single nucleotide variant analysis and the date of strain isolation. Colours indicate the case’s location at the time of the predicted exposure. Dashed arrows indicate transmissions that were not supported by the other investigation method. SNV, single nucleotide variant

Transmission events using a standard outbreak investigation [4] versus WGS-generated chain of transmission are found in Fig. 1. Of the 27 transmission events determined using a traditional investigation strategy, 12 (44.4%) were identical to transmission events predicted by WGS, while 15 (55.5%) were discordant (Fig. 1b).

In the standard investigation, patient 2 was predicted to be the central source for the two principal transmission arms (Fig. 1a). The cluster of transmission that occurred on Medical Ward (MW) 1 in the first quarter of 2013 (patients 6–9) was believed to have been introduced by patient 2. WGS analysis, however, did not support this conclusion and suggested that the strain from patient 6 was more closely related to patient 5 than patient 2 (1 SNV vs 3 SNV, respectively). Similarly, the cluster on MW 2 in 2013 (patient 17–24, and 26) was initially believed to have originated from patient 8, whereas WGS suggested that the strain in this cluster was identical to the strain of patients 3 and 5 (0 SNV) and different from patient 8 (2 SNVs). Using WGS, patient 3 was deemed central to the outbreak, contributing both to clusters on MW 1 in early-to-mid 2013 and the cluster on MW 2 in late 2013 (Fig. 1b). On the other hand, the WGS-generated chain of transmission also incorrectly identified some transmission events. For example, the source of patient 17 was attributed to patient 5 by WGS and date of sampling, but considering that the strains of patients 3, 5 and 17 were identical, the source of patient 17 may have been patient 3 or an unknown intermediate case. In fact, spatiotemporal analysis indicated that patients 3 and 17 were both temporally located on MW 1 prior to patient 17 being transferred to MW 2 where the outbreak strain rapidly disseminated. Other notable discrepancies between both investigation methods included transmissions to patients 6, 10, 11, 13 and 28 (Fig. 1).

In the standard investigation, 26 of the 28 cases were found to have shared a ward with a potential source case. [4] In the WGS-generated chain of transmission, 23 of the 27 transmission events could be explained by patients sharing a ward with a known case, though acquisition of the outbreak strain occasionally occurred on a different ward than previously suspected (Fig. 1b).

Two discordant events differed only in terms of directionality. Using standard epidemiologic tools, patient 2 was thought to have transmitted the outbreak strain to patient 3, and patient 13 to 14. However, WGS suggested that the opposite had actually occurred, postulating that patients 3 and 14 had either acquired the outbreak strain at an earlier date than originally thought or that there were undetected carriers.

SNV differences between transmission events in the standard epidemiologic investigation ranged from 0 to 4 SNVs, while the range was from 0 to 2 SNVs in the WGS-generated chain of transmission. The largest SNV difference between the index case and a subsequent case was 19 SNVs in the standard investigation (index case and patient 28; Fig. 1a) compared to 5 SNVs in the WGS investigation (index case and patients 13, 15 and 16; Fig. 1b). The average number of SNVs (standard deviation) per transmission event was 1.63 (1.31) and 0.63 (0.79) for the standard vs. WGS-generated investigations, respectively (*p* = 0.001 by Student’s T-test).

### Comparison of conventional typing by PFGE and genomic analysis

Four pulsovars were identified by PFGE. By WGS, patients 3, 5, 17, and 19 to 23 had no SNV differences. However, PFGE established different band patterns, initially grouping the isolates into pulsovar A5 or A5-a. Examination of these sequences identified three small DNA segments unique to the earlier isolates, but none which explained the band variation seen on PFGE. It is suspected that a genetic event (insertion, deletion or recombination) occurred at or near the restriction site for *Apa*I causing different band numbers or shifting of band sizes. SNV analysis may not have detected this change as it may have occurred at a site of multiple events outside of our SNV parameters (i.e. 96.8% of core genome covered by WGS) or was a large event not captured by the SNV workflow. Similarly, PFGE grouped isolates 13 and 15 into pulsovar A4, and isolate 16 to pulsovar A6, but there were no SNV differences between these 3 strains by WGS.

## Discussion

Outbreak investigations typically rely on spatiotemporal data and PFGE, but WGS is also becoming increasingly used. Our study compared the chains of transmission of a CPAb outbreak using a standard epidemiologic investigation and a WGS-based technique. Using qualitative and quantitative comparative strategies, our investigation identified many similarities between both investigations, but also major differences. Nearly 50% of the predicted transmissions were identical between both methods, and upon review of the epidemiologic data it was found that several transmissions had occurred on different hospital wards than previously assumed.

The extent of these differences was unexpected. Some discrepancies may be explained by the fact that transmission events in standard epidemiologic investigations are typically deduced based on commonality between a source and a case and are prone to missing transmission events between patients with little spatiotemporal commonality [5, 21]. The location where transmission occurred could not be identified for four patients from the WGS-generated chain after review of the epidemiologic data; these transmission events were at risk of being missed using traditional tools. Additionally, PFGE detected different band patterns among genetically related isolates by WGS, likely influencing some of the dissimilar epidemiologic predictions. Overall, the chain of transmission determined by WGS might be perceived as more accurate due to the close genetic relatedness of the strains. However, some WGS-generated transmission events may be incorrect: upon reviewing spatiotemporal data, the origin of patient 17 is probably patient 3 instead of patient 5.

Both chains did retain an important degree of concordance, which is noteworthy considering that the WGS-generated chain of transmission was fully blinded to any spatiotemporal information and to the PFGE typing results. It provides a glimpse of the potential for automated outbreak investigations. Some transmission events determined by traditional method were shown to be implausible by WGS given the high number of SNVs between the source and the recipient. However, it can be argued that some discrepancies are of minor importance, such as linking cases 19–23 to either patient 17 or 18. Knowing these differences would have changed little in terms of outbreak confinement measures.

Still, some observations suggested that WGS may have had practical value had it been available. Firstly, the WGS investigation suggests that infection control strategies around patient 3 had failed, leading to the cluster of cases on MW 2. Patients 3 and 17 had been hospitalized on the same ward prior to 17 being diagnosed with the outbreak strain, but this association was not captured in the original investigation. Had it been, there could have been efforts to understand why control measures were breached. Secondly, the SNV analysis suggested missed opportunities for earlier case findings. Real-time WGS-assisted investigation could have suggested the presence of additional unidentified cases. Finally, it was unclear where four cases acquired the outbreak strain. Had the connection between source and case been made in real-time, it might have assisted in generating hypotheses regarding transmission.

WGS is already used for public health surveillance in various jurisdictions [22–25], and has helped perform accurate contact tracing in health-care settings [11, 26, 27]. Our findings further support the potential role of WGS in hospital outbreak investigations, particularly as the technology becomes more affordable and accessible [6, 8, 28, 29].

Two additional observations are noteworthy. Firstly, our investigation demonstrated surprising discrepancies in the phylogenetic assessment of clonality between PFGE and WGS. While PFGE occasionally lacks discriminatory power with closely related bacterial strains [6, 30, 31], it seemed unusual for it to assign different pulsovars to strains that were found to have no SNV differences. However, *A. baumannii* can undergo significant horizontal gene transfer and genetic recombination, even over short time periods [32]. Other studies have observed low discriminatory powers of PFGE when applied to *A. baumannii* [27, 33]. With *A. baumannii*, PFGE may be too discriminatory between isolates and distort the interpretation of phylogenetic differences, essentially leading to “false-negative” results where WGS data may provide indistinguishable differences [7]. SNV analyses may be superior to PFGE for *A. baumannii* typing as it ignores changes due to horizontal gene transfers and genomic recombination.

Secondly, this study represents the third reported identification of the acquired class D carbapenemase *bla*_OXA-237_, and its first identification in Canada. The *bla*_OXA-235-like_ subclass, which includes *bla*_OXA-237_, was first recognised from *A. baumannii* isolates in the United States and Mexico in 2013 [34] and was implicated in a multi-center outbreak in Oregon between 2012 and 2014 [35]. This resistance gene was located on a plasmid found in the most prominent *A. baumannii* clonal group, representing a potential for extensive dissemination [36].

Our study has some limitations. Due to its retrospective nature, it remains unknown whether performing WGS in real-time would have altered the outbreak progression. Despite all the discrepancies observed, the standard epidemiologic investigation was ultimately successful in controlling the outbreak. Also, review of the available epidemiologic data could not account for the method of CPAb acquisition in four cases. Subsequent epidemiologic investigation was not conducted to link these cases with a source. *A. baumannii* can contaminate the hospital environment, such that transmission through fomites or health care workers is possible [3, 27]. Neither investigation can account for the presence of additional unidentified colonized patients [37]. Finally, the lack of a standardized definition for “bacterial clones” in molecular epidemiology for *A. baumannii* also complicates WGS investigations [7, 33, 38–40]. Bacteria naturally undergo genomic diversification, and SNVs can accumulate at a rate of 2–10/year in most bacterial genomes [7, 27]. Still, our data demonstrates minimal genetic diversification between isolates, with no more than 5 SNV differences from the index case and some with 0 SNV changes over nearly a year.

## Conclusion

This study suggests that an investigation based on WGS and date of sampling was able to create a transmission sequence that was in many aspects similar to a traditional outbreak investigation and that detected transmission events that had not been suspected using traditional tools. WGS may help improve the understanding of transmission during outbreaks when used alongside epidemiologic data and clinical information.

## Abbreviations

CPAb: Carbapenemase-producing *Acinetobacter baumanii*
DNA: Deoxyribonucleic acid
MALDI-TOF MS: Matrix-assisted laser desorption/ionization time-of-flight mass spectrometry
MW: Medical ward
PFGE: Pulse-field gel electrophoresis
SNV: Single nucleotide variant
WGS: Whole genome sequencing
